# Current Studies of Immunotherapy on Glioblastoma

**Published:** 2014-04-05

**Authors:** Neena Stephanie Agrawal, Rickey Miller, Richa Lal, Harshini Mahanti, Yaenette N. Dixon-Mah, Michele L. DeCandio, W Alex Vandergrift, Abhay K. Varma, Sunil J. Patel, Naren L. Banik, Scott M. Lindhorst, Pierre Giglio, Arabinda Das

**Affiliations:** 1 Department of Neurosciences (Divisions of Neurology and Neurosurgery) & MUSC Brain & Spine Tumor Program Medical University of South Carolina, Charleston, SC 29425, USA; 2 Ralph H. Johnson VA Medical Center, Charleston, SC, USA

## Abstract

Glioblastoma is a form of brain tumor with a very high morbidity and mortality. Despite decades of research, the best treatments currently in clinical practice only extend survival by a number of months. A promising alternative to conventional treatment for glioblastomas is immunotherapy. Although proposed over a century ago, the field of cancer immunotherapy has historically struggled to translate it into effective clinical treatments. Better understanding is needed of the various regulatory and co-stimulatory factors in the glioblastoma patient for more efficient immunotherapy treatments. The tumor microenvironment is anatomically shielded from normal immune-surveillance by the blood-brain barrier, irregular lymphatic drainage system, and it’s in a potently immunosuppressive environment. Immunotherapy can potentially manipulate these forces effectively to enhance anti-tumor immune response and clinical benefit. New treatments utilizing the immune system show promise in terms of targeting and efficacy. This review article attempts to discuss current practices in glioblastoma treatment, the theory behind immunotherapy, and current research into various clinical trials.

## Introduction

Glioblastoma, the most frequent and malignant primary brain tumor, stands apart from other neoplasms by its biology and location within the central nervous system (CNS) [1]. In spite of aggressive multimodal treatment including surgical resection, radiation therapy, and cytotoxic chemotherapy, the disease remains incurable with a 2-year survival rate of 26.5% [2]. The failure of conventional oncologic treatment to selectively target glioblastoma cells has prompted investigators to look for new and more targeted therapeutic options as well as prognostic biomarkers that will help us better understand the variation of outcomes. It is clear that new approaches for developing effective and targeted treatment options are needed for patients with glioblastomas. Neurologists and neurosurgeons provide reports that glioma patients who suffer postoperative infections near the tumor bed seem to do better than the average patient similar to the observations made over a century ago [3]. The infection group had a significant advantage in median survival (30 months compared to 15 months) in the non-infected tumor patients. A higher CD4+ counts leads to a significantly longer median survival rate (19.7 months) when compared to a lower CD4+ counts (13.1 months) in patients [4]. All of these observations suggest a strong rational to use immunotherapy for glioblastoma patients. Immunotherapy offers a different mechanistic approach from chemotherapy, targeted therapy, radiation, and surgery. Recent success in the treatment of other cancers has fueled a resurgence of interest in this approach [5]. Currently, there are more than 20 FDA approved immunologic products used in treatment of human malignancies [6]. The sevaccine approaches to elicit strong specific immune responses to tumor antigens, approaches involving adoptive transfer of in vitro expanded, naturally arising, or genetically engineered tumor-specific lymphocytes, therapeutic administration of monoclonal antibodies to target and eliminate tumor cells, and approaches that inhibit or destroy the molecular or cellular mediators of cancer induced immunosuppression such as CTLA-4, PD-1, or Treg cells [7]. Unfortunately, these efforts have been unsuccessful in most of other cancers. This may be due to the lack of understanding in immunology of glioblastoma. A major potential pitfall for immunotherapy in glioblastomais due to a number of factors: relative immune-privilege of the brain, this may be due to the blood brain barrier, low numbers of T lymphocytes, and lack of lymphatic systems which makes it challenging for immune cells to enter the CNS [8]. Patients with glioblastoma exhibit a relative systemic immune suppression compared to the general population. The tumor microenvironment is rich with immunosuppressive factors secreted by the tumor like transforming growth factor beta (TGF-*β*) and vascular endothelial growth factor (VEGF) [9]. It is well known that these factors suppresses T cell proliferation and cytotoxic function by inhibiting dendritic cell (DC) maturation, diminishing absolute counts of CD4+ T cells, and also increased fraction of T-regulatory cells (T-regs) [10]. High proportions of T-regs actively inhibit conventional CD4+ T cells, CD8+ T cells, DCs, and NK cells thus dampening immune responses around tumors [11].

Cells of myeloid lineage have been increasingly associated with immunosuppression in a number of systems. Myeloid-derived cells at different states of maturation have been studied as potent inactivators of both CD4+ and CD8+ T cells and thus may possess immunosuppressive abilities [12]. Another recent study has shown that exposing glioblastoma cells to IFN-*γ* decreased TGF-*β* expression but increased expression of PD-1 ligand and Indoleamine-2, 3-Dioxygenase (IDO) [13]. It is reasonable to speculate that other immunosuppressive cytokines exhibit comparably complex interactions. Thus, it is important to understand the role of immunosuppression parameters and factors in tumor progression in patients with glioblastoma. In future immunotherapy, the immune suppression by allowing effective immune targeting of gliomass of those patients with glioma might have less tumor progression and improved outcomes [14]. In a significant number of glioblastoma patients, the blood brain barrier is disorganized by single or combined chemotherapy or radiation which leads to breakdown of the tight junctions between endothelial cells that facilitate migration of leukocytes into the CNS [15]. In that case, activated T cells that encounter their antigen are retained in the CNS. Human leukocyte antigen (HLA) presentation occurs on astrocytes, microglia, and endothelial cells which are essential elements for immune function [16]. The net balance is that CNS immune surveillance still occurs. In spite of these apparently local as well as global aberrations in cellular immunity, most of the glioblastoma patients are generally not systemically immune compromised prior to the growth of their tumor [12,17]. It is therefore likely that tumor -associated immunosuppressive factors will similarly affect clinical attempts to augment antitumor responses.

Therefore, targeting tumor-associated immunosuppression in glioblastoma patients will be critical for the development of meaningful immunotherapeutic strategies. Immunization against glioblastomas can occur in the form of passive or active immunotherapy [3,18,19]. Active immunotherapy provides a boost to the patient’s native immune system (including peptide based therapy utilizing MHC class I molecules and cell based therapy utilizing DCs) by priming it with antigen exposure. By contrast, in passive immunotherapy, a patient is given immune cells or antibodies capable of targeting the tumor cells [19]. Passive immunotherapy does not require activation of the patient’s own immune system, but instead immune cells are active in different ways. Immune cell activity takes place in the following ways: (1) the direct injection of monoclonal antibodies (ex. bevacizumab is a humanized IgG1 monoclonal antibody), (2) stimulation of the immune system with cytokines (Ex. IL-2), and (3) treatment with stimulated immune effector cells by adoptive immunity or cell-based therapy immunotherapy [18,20]. In adoptive immunity, immune cells (lymphocyte-activated killer cells: LAK and cytotoxic T lymphocytes: CTL) activated ex-vivo are administrated to the patient either by systemic injection or directly into the tumor or tumor resection cavity [20,21]. LAK cells are generally obtained by cultivating autologous peripheral lymphocytes in the presence of IL-2, which yields both T and NK cells. The immune reaction provided by LAK cells is non-specifically cytotoxic and is largely not tumor-directed [22]. By contrast, collecting peripheral blood mononuclear cells or tumor infiltrating lymphocytes and then stimulating them ex vivo with antigens prepares CTLs in a tumor-directed fashion [23]. Thus, for glioblastoma immunotherapy, autologous tumor cells are also used for the antigen stimulation thus yielding CTLs that have been activated.

## Current Research in Glioblastoma Immunotherapy

**Figure 1:**
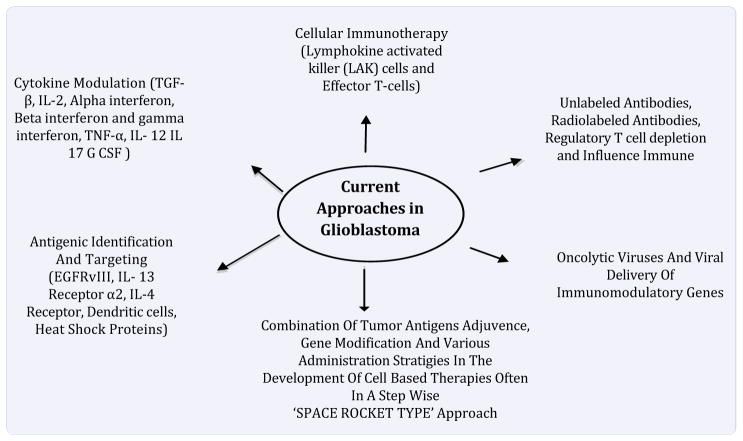
Schematic diagram of current immunotherapy’s in glioblastoma

The field of immunotherapy as it is applied to glioblastoma is wide and varied (Figure 1). Although immunotherapeutic approaches have met with mixed success so far, immunotherapy continues to be actively pursued because of its potential to attack infiltrating, high-grade gliomas. Recently, clinical trials demonstrated that using infusion of activated autologous immune cells or active immunotherapy with tumor antigens and dendritic cells successfully induced anti-tumour immunity and some radiological responses [24]. However, large randomised trials are still needed to prove the usefulness of this immunotherapy in brain tumors.

Currently, most drugs under investigation as immunotherapeutic agents depend on designing and confirming immunotherapy in the existing pre-clinical glioblastoma models: both immunocompetent models (normal immune system) and immunodeficient models, which lack specific immune-related molecules. To properly utilize immunocompetent models in an orthotopic context, implantation of genetically compatible tumor cells is required to prevent graft vs. host immunity. This would be analogous to implanting tumor cells derived from a donor C57BL/6 mouse into a host C57BL/6 mouse, which is useful for studying how immune cells infiltrate, respond to, and mediate anti-tumor immunity [25,26]. However, a different approach can be used to study immunotherapy by utilizing an immunodeficient model acting as a litmus test to determine whether a particular therapy requires a specific immune molecule or cell type to mediate an anti-tumor effect. This approach is useful for researchers to understand the mechanism of action for immunotherapy in a brain tumor model that has a T-cell deficiency. This model is more advantageous because it has the ability to isolate which would lead to an unproductive investigation and that T cell functionality is required for translating this therapy into patients with brain tumors.

Our groups and others have shown that all trans retinoic acid (ATRA) can modify the immunogenicity of tumor cells both in vitro and in vivo through differential regulation of MHC class I and intercellular adhesion molecule-1 (ICAM-1) as well as increase the sensitivity of glioblastoma to NK-cells [27,28]. These results suggest that tumor cells can be converted to efficient antigen presenting cells for direct antigen presentation and T-cell stimulation. It has been shown by our group that IFN-*γ* is an important biomolecule for positive regulation of the MHC presentation machinery [27]. The treatment of glioblastoma cells with IFN-*γ* induces apoptosis and the extent of cell death is enhanced by pretreatment with ATRA. It was also shown that a combination of ATRA and IFN-*γ* expressed higher levels of HLA class II and HLA-DM molecules in glioblastoma T98G and U87MG cells than IFN-*γ* alone suggesting that the combination of ATRA with IFN-*γ* may overcome the defect in class II-mediated immune recognition of glioblastoma. Recent studies of human glioblastoma tissue samples have reported tumor-infiltrating lymphocyte populations significantly enriched for T-regulatory cells (Tregs), which are a CD25+, FoxP3+, and subset of CD4+ helper T cells, which suppress immune activation through interactions with T cells, B cells, NK cells, DCs, and macrophages [13]. Tregs have been shown to express CTLA-4, which decrease the secretion of cytokines (IL-2 and IFN-*γ*), and also skew the immune response away from a cytotoxic Th1-mediated response in favor of a Th2 response [13]. Glioblastoma cells also appear to secrete high levels of CCL22 and CCL2, as compared to low gradeglioma, which facilitates Treg trafficking to the tumor [13]. These observations have led to interest in developing immunotherapies for glioblastoma that target Tregs. Currently, STAT3 inhibitor WP1066, and blocking antibodies against CTLA-4 and CD25, has been shown to decrease Treg proliferation [29]. A new approach being evaluated in clinical trials involves the use of monoclonal antibodies to block immunosuppressive molecules such as CTLA-4 or PD-1 expressed by T cells. The effectiveness of monoclonal antibodies that block the PD-1 ligand, PD-L1, which can be expressed on tumor cells and normal host cells, is also being explored [30]. A recent phase III clinical trial reported that therapy with CTLA-4–blocking antibodies imparted a significant survival benefit in approximately 30% of patients with other cancer, making this drug a promising treatment for glioblastoma. The success of glioblastoma clinical trials will encouraged interest in blocking other potential effectors of immunosuppression including the soluble (such as IDO and TGF- β) and cellular (such as Treg cells and MDSCs) mediators of the process. Undoubtedly, there is much to be learned about the benefits and risks of inhibiting the different immunosuppressive mechanisms including TGF-β that may be simultaneously operating in the glioblastoma patient [14,31,32]. The involvement of TGF-β in multiple tumorigenic pathways, which promote tumor growth and invasion by sustaining glioblastoma stem cells, promoting angiogenesis, and up regulating MMP-2 expression, makes this cytokine an enticing target for immunotherapy. TGF-β also promotes immunosuppression in glioblastoma by inhibiting T cell activation and proliferation, blocking IL-2 production, suppressing activity of NK cells, and promoting Treg activity. Current approaches to IL-2 and beta interferon (IFN-β) have been extensively studied in cancer immunotherapy either alone or combination with temozolomide [13]. A more recent trial of IFN-α in combination with local BCNU, a chemotherapeutic related to lomustine (CCNU) and semustine, which partially overlaps the activity/toxicity of alkylating agents, delivery in patients with recurrent glioblastoma reported a 6-month progression-free survival in 2/9 patients [13]. These results confirmed by a study, which demonstrated that mice deficient in type 1 interferon and induced to develop gliomas de novo via p53 knockdown exhibited enriched populations of tumor infiltrating myeloid-derived suppressor cells and Tregs, as well as a decrease in the number of tumor-infiltrating CD8+ T cells. Recently, granulocyte-macrophage colony-stimulating factor (GM-CSF) was used as an adjuvant in a phase II vaccination study of patients with newly diagnosed glioblastoma.

The proposed mechanism of action was GM-CSF promotion of CD8+ cytotoxic T cell response when combined with antitumor vaccines [13,33,34]. The IL-13R *α* 2 antigen and IL-4 receptor (IL-4R) are also promising targets for immunotherapy because they are highly expressed on glioma cells but not on host CNS cells [13,35]. Subsequent clinical trials of fused protein (IL-13-PE38QQR and IL-4-PE38KDEL) using the same construct with stereotactic injection as the delivery method, showed similar findings of safety and efficacy.

One well-studied technique is the development of monoclonal antibodies that target specific receptors that are unique to tumor tissue. One such candidate is the vascular endothelial growth factor receptor (VEGF-R). Many of the morbidities associated with glioblastoma are associated with the edema surrounding the primary tumor. This edema is largely produced secondary to disruption of the blood brain barrier and to the production of new vasculature mediated by VEGF released by the tumor [15,13]. Drugs targeting the VEGF pathway have the benefit of reducing edema therefore potentially reducing morbidity and halting further tumor growth. The most successful agents developed thus far have all been monoclonal antibodies. The most well known is bevacizumab, which was approved for use in recurrent glioblastomamultiforme in 2009. In patients with recurrent glioblastoma, patients receiving bevacizumab had6-month progression-free survival rates of 42.6%, and patients receiving combination bevacizumab and irinotecan had survival rates of 50.3%. Both of these rates are significant improvements over prior figures of 9 to 21% [36]. However, relapses still occur despite these advances and other drugs targeting this pathway are being developed. Aflibercept is a drug that binds to VEGF and placental growth factor. In a phase II trial, 19 out of 26 glioblastomapatients with reduced blood levels of VEGF and various cytokines had at least some radiographic improvement in tumor burden after treatment with aflibercept [37]. Antibodies targeting other molecules in the VEGF pathway, such as VEGFR-1,2,3, platelet-derived growth factor, epidermal growth factor, and placental growth factor are also under development in ongoing clinical trials [38].

One problem with targeting growth receptors is that these therapies are not specifically targeted at tumor cells. Damage to healthy brain tissue caused by these agents can result in significant morbidity and, in some of the most serious cases, an allergic encephalomyelitis. Therapies that utilize T cells inoculated against tumor antigens also suffer from this complication due to the fact that many of the anti-tumor antigens produced are shared with normal cells. However, a tumor-specific antigen for glioblastoma has been recently identified. Epithelial growth factor variant III (EGFRvIII) is a common variant that is characterized by an 801 base pair in-frame deletion that causes a split in amino acids 6 and 273. A glycine is inserted between amino acids 5 and 274 [39]. This new arrangement causes the tyrosine kinase domain to be constitutively activated resulting in increased tumorgenicity and resistance to chemotherapy and radiation treatment. This mutation is fairly common about 40% of glioblastomas show EGFR gene amplification and 67% of these have been found to carry the EGFRvIII mutation [40]. These characteristics of the EGFRvIII mutation provide a tumor-specific target that is found with high frequency in malignant gliomas. Preclinical studies on this variant have shown that murine models produce an antibody response to the EGFRvIII-specific peptide PEPvIII. This is a 14-amino acid peptide representing the unique region of EGFRvIII that is conjugated to keyhole limpet hemocyanin (PEPvIII-KLH) [41].

Murine intra-cerebral melanoma models inoculated with the PEPvIII-KLH vaccine along with dendritic cells were found to have developed a humoral response against the variant composed of IgG1 and IgG2a class antibodies. In addition, the presence of antibodies was directly correlated with clinical response and regression of tumor without autoimmunization against the CNS [42]. In humans, phase I and II clinical trials have been concluded. The phase I trial VICTORI showed that patients with glioblastoma could safely be treated with the vaccine composed of dendritic cells inoculated with PEPvIII-KLH [43]. These patients also had a longer survival rate when compared to equivalent patients in other published data. The phase II activate trial endeavored to assess the immunogenicity of the vaccine and progression-free survival in patients with newly diagnosed EGFRvIII-expressing glioblastoma. After the elimination of ineligible patients, 18 patients were included in the trial starting 4 weeks after their last radiation treatment. The first three vaccinations were given every two weeks and were subsequently spaced out to every month until there was radiographic evidence of tumor progression or death. The median progression-free survival for these patients was 14.2 months compared to 6.3 months in the matched cohort. Survival time was also extended in trial patients to 26.0 months compared with 15.0 months in the matched cohort [44]. Currently, phase III trials are underway [45].

Related to the PEPvIII-KLH vaccine is vaccination with dendritic cells by themselves. Normally, dendritic cells exist in most tissue types in an immature state, sampling potential antigens. When dendritic cells present their antigens, they are able to activate both CD4+ and CD8+ cells, a process that is essential for effective cell-based immunity [46]. In addition, dendritic cells have been shown to activate natural killer (NK) cells, providing a powerful method for eliminating glioma cells that do not express MHC-1 molecules on their surfaces [47]. The principle behind a dendritic cell vaccine involves activating dendritic cells removed from a patient with antigens that are tumor-specific, then re-introducing them to the patient. Antigens that have been favored for vaccine production are usually whole tumor-cell antigens, and are isolated in a variety of ways, including acid elution of membrane proteins, various lysates, gamma-irradiation, and isolation of protein from paraffin-embedded samples [48]. Over the last decade there have been a variety of clinical trials demonstrating the efficacy of various dendritic cell vaccines. One recent clinical trial by Chang et al. used a vaccine prepared by using a lysate digested with collagenase. The patients in this trial were treated with the vaccine following surgery and subsequent radiotherapy. They received the vaccine subcutaneously in axillary lymph nodes once a week for 4 weeks, then every 2 weeks twice, then finally monthly 4 times, totaling 10 doses. The 16 patients who completed the trial had a median survival of 525 days and 5-year survival of 18.8%. This was compared to 63 historical control patients with a median survival of 380 days and 5-year survival of 0%.

Interestingly, the authors of the study found that patients with relapsed glioblastoma responded better to the treatment than did newly diagnosed patients. Side effects of this treatment were minorand included transient AST/ALT elevations [49]. Many more trials testing various dendritic cell vaccines are currently being done.

One particularly creative avenue of research is the use of oncolytic virus therapy to treat glioblastoma. This technique utilizes oncolytic viruses that are modified so that they selectively infect tumor cells while ignoring normal cells. Glioblastoma is ideal for such a technique due to its relatively isolated tissue of origin and its lack of propensity towards metastasis. Plus, normal brain tissue is static in the post-mitotic phase, making it less of a target for viruses, which require actively dividing cells to propagate [50]. Human viruses used for this technique must be modified so that they do not infect normal tissue. The most successful of these have been herpes simplex virus (HSV) and adenovirus. HSV is a DNA virus with an especially large genome, allowing for the addition or removal of relatively large genes without disruption of viral replication. The first mutant used against gliomas was G207, containing deletions in both gamma (1) 34.5 loci and a disabling insertion of lacZ in the UL39 gene, resulting in a virus that is unable to replicate in normal brain tissue, but proliferates in glioma tissue [51]. A phase I trial using this variant in 21 patients showed that the virus could be administered without the development of encephalitis [52]. A subsequent phase Ib trial also demonstrated safety of administration along with some initial evidence of viral replication in several of the six patients, though the results were not uniform [53]. In light of these and similar results using other modified herpes viruses, oncolytic strains carrying cytokines and other immunostimulatory agents have been developed. So far the G207 variant modified to express murine IL-12 has shown some effect in activating primate lymphocytes in preclinical trials [54]. Techniques utilizing adenovirus include disruption of the E1A and E1B regions of the viral genome, without these regions adenovirus can only infect and replicate in tumor cells that have defective cell cycle regulation through disrupted retinoblastoma protein and protein 53 tumor suppressors [55]. So far only preclinical trials and one phase I trial have been completed in this area, though initial results are promising [56]. In addition to human viruses, vaccine viruses and non-human viruses have been considered as possible oncolytic viruses. The benefit to vaccine viruses is that they have already been inactivated for normal tissue and tend to be less pathogenic, only requiring modification to ensure activation against tumor cells. Promising candidates have been vaccines against measles, polio, and rabies. Non-human viruses are also an interesting avenue of research due to the fact that they do not normally infect healthy cells and must be modified specifically in order to infect tumor cells. Currently, most research on vaccine viruses and non-human viruses remains in the preclinical stage [49]. Recent approaches include: (1) use of autologous tumor transfected with cytokine genes to express cytokine or DC-tumor cell fusions; (2) vaccination with a heat shock protein in complex with autologous tumor derived peptides; (3) delivery of autologous tumor cells via a viral vaccine vector using Newcastle Disease Virus (NDV). NDV offers the advantage of a single stranded RNA virus that poses little health hazard to humans and has the ability to selectively kill human tumor cells.

## Future Directions

The future of immune therapies in glioblastoma involves challenges related to enhancing antigen presentation capabilities, effectively breaking tumor-induced immune tolerance, improving a strong and long lasting anti-tumor T cell activation of tumor-specific cytolytic effector cells, and the standardization and upscale production of cell based therapy. Similarly, more clarification is required as to when and how immune therapy should be given with other modalities. The role of steroid and DC based immunotherapy use in this population of patients will require well-designed and appropriately powered clinical trials. There are a few potential targets that could enhance the immune system’s recognition of the tumor. One strategy might be to deplete the regulatory T cells. Once T cells are activated, they up regulate molecules such as CTLA-4 and PD-1 to limit their activity. Use of blocking humanized monoclonal antibodies to these checkpoint molecules appear very promising and have already made it to the clinic in treating patients. To that end, an antibody against CD4 or CD25 could be used to target Tregs, or more general immunotoxins could be used. A phase I clinical trial using a vaccine comprised of autologous tumor cells genetically modified by transforming growth factor–*β*2 (TGF- *β*2) antisense vector in 6 patients with recurrent glioblastoma was well tolerated with indications of anti-tumor induced immunity. In the future, we can use this approach to treat glioblastoma patients. Use of additional inflammatory cytokines such as IL-12, IL-7, and IL-15, activating antibodies to co-stimulatory molecules such as CD40, or blocking antibodies to immune inhibitory cytokines such as IL-10 or TGF- *β* in combination with DC vaccination, can potentially enhance clinical activity which of these strategies in combination with vaccination that will yield the best therapeutic ratio (most effective and less toxic) is still to be determined. The rational to give chemotherapy with immunotherapy may relate to the chemotherapeutic effects on tumor release of relevant antigens, on inhibiting the regulatory compartment, and on the ability to change the tumor vasculature providing better access for effector cells. Another possibility is that vaccination sensitizes the tumor to chemotherapy.

## Conclusion

Research over the last 20 years has demonstrated that immune therapy for glioblastoma triggers a measurable immune response in spite of poor tumor antigenicity and considerable immune suppression. The challenge of immunotherapy is to understand the various regulatory and co-stimulatory factors in the patient and the tumor microenvironment, and then manipulating these forces effectively to enhance anti-tumor immune response and clinical benefit. Interpretation and comparison of the results of clinical trials using immune therapy against glioblastoma is extremely difficult because of a number of reasons: (1) heterogeneity in study design, therapeutic approach used, immune endpoints measured, and patient eligibility criteria; (2) The limited number of patients with glioblastoma, the lack of a cooperative group that can do large clinical trials for the study of brain tumor immunotherapy, and the variability in approaches and immune monitoring assays used are the major barriers to determine if immune therapy could be part of the standard of care; (3) The classic design of cancer clinical trials does not fit the immune therapy model as few phase I/II clinical trials published are not randomized and use historic controls to compare outcomes; (4) Most clinical trials included patients with recurrent glioblastoma, who may have a poor functional status, large tumor burden and been heavily pre-treated making less likely to benefit of immune therapy; (5) Furthermore, some trials include patients with newly diagnosed and recurrent disease, and on occasions, include patients with anaplastic gliomas. Surrogate endpoints, like immunologic assays and brain imaging studies, have not been harmonized and validated in most cases. Thus, there is need for harmonization and validation of immunologic endpoints as well as imaging techniques that allow adequate monitoring of patients with brain tumors receiving immune base therapies. With our expanded knowledge of immune pathways and the effects tumors have on immune function, more novel and effective strategies can be developed in the future.

## Figures and Tables

**Table 1:** Ongoing Clinical Trials

Trial Type Description		Clinicaltrials.gov Identifier	Status
Phase I	Tumor Associated Antigen pulsed dendritic cell vaccine	NCT00576641	Completed
Phase I	Cell-based immunity Autologous Lymphoid Effector Cells Specific Against Tumour cells (ALECSAT)	NCT01588769	Completed
Phase I	Intratumoral infusions of a CD8+ cell line expressing IL-13-Zetakine and HyTK with IL-2	NCT01082926	Recruiting
Phase I/II	Efficacy of basiliximab in patients in conjuntion with TMZ and other immunotherapy	NCT00626483	Active
Phase II/III	Proteome-based personalized immunotherapy using hematopoietic stem cells	NCT01759810	Active
Phase II	Cellular therapy of GBM with IL-2-stimulated lymphoctes	NCT00331526	Completed
Phase II	Tumor lysate-pulsed dendritic cell vaccine	NCT00576537	Completed
Phase II	Immunostimulating agent CpG-ODN	NCT00190424	Completed
Phase III	Tumor lysate antigen-pulsed autologous dendritic cell vaccine	NCT00045968	Recruiting
Phase I	Alloreactive cytotoxic T lymphocytes and IL-2	NCT01144247	Recruiting
Phase II	Synthetic peptide-pulsed dendritic cell vaccine	NCT01280552	Active
Phase I	Adenoviral vector containing herpes simplex thymidine kinase gene plus valacyclovir	NCT00751270	Active
Phase II	Adenoviral vector containing herpes simplex thymidine kinase gene plus valacyclovir	NCT00589875	Active
Phase I/II	PEP-3-KLH conjugate vaccine and daclizumab	NCT00626015	Active
Phase I	Cytomegalovirus pp65-LAMP mRNA-loaded dendritic cell vaccine	NCT00639639	Active
Phase I	Brain tumor stem cell mRNA-loaded dendritic cell vaccine	NCT00890032	Recruiting
Phase II	PEP-3-KLH conjugate vaccine	NCT00643097	Active
Pilot	Expanded autologous CD8+ T-cells expressing IL-13 zetakine receptor and HyTK protein	NCT00730613	Completed
Phase I	Allogeneic brain tumor stem cell-loaded dendritic cell vaccine	NCT01171469	Active
Phase II	Autogeneic glioma stem-like cell (A2B5+)-loaded dendritic cell vaccine	NCT01567202	Recruiting
Phase II	Autologous dendritic cell vaccine	NCT00323115	Active
Phase I	Vorinostat combined with irinotectan and bevacizumab	NCT00762255	Active
Phase II	TVI-Brain-1 T-cell vaccine	NCT01290692	Active
Phase I/II	Anti-EGFRvIII chimeric antigen receptor-expressing T cells	NCT01454596	Recruiting
Phase II	CDX-110 with GM-CSF vaccine	NCT00458601	Active
Phase I	Tumor peptide-based glioma vaccine	NCT01403285	Recruiting
Phase I	Autologous tumor lysate-pulsed dendritic cell vaccine	NCT00068510	Active
Phase II	Autologous tumor lysate-pulsed dendritic cell vaccine	NCT01204684	Recruiting
Phase I/II	Tumor stem cell derived mRNA-transfected dendritic cell vaccine	NCT00846456	Active
